# Accuracy (trueness and precision) of 3D printed orthodontic models finalized to clear aligners production, testing crowded and spaced dentition

**DOI:** 10.1186/s12903-023-03025-8

**Published:** 2023-06-02

**Authors:** Vincenzo Grassia, Vincenzo Ronsivalle, Gaetano Isola, Ludovica Nucci, Rosalia Leonardi, Antonino Lo Giudice

**Affiliations:** 1 Multidisciplinary Department of Medical-Surgical and Dental Specialties School of Medicine and Surgery, University of Campania Luigi Vanvitelli, Naples, Italy; 2 Department of General Surgery and Medical-Surgical Specialties, Section of Orthodontics, University of Catania, Catania, Italy

**Keywords:** Orthodontics, Digital orthodontics, Clear aligners, 3D printing

## Abstract

**Background:**

The study's objective was to assess the accuracy (trueness and precision) of orthodontic models obtained from crowded and spaced dentition finalized for the production of clear aligners. Four 3D printers featuring different technologies and market segments were used for this purpose.

**Methods:**

Two digital master models were obtained from two patients featuring respectively crowded dentition (CM group) and diastema/edentulous spaces (DEM group). The 3D printers tested were: Form 3B (SLA technology, medium-professional segment), Vector 3SP (SLA technology, industrial segment), Asiga Pro 4K65 (DLP technology, high-professional segment), and Anycubic Photon M3 (LCD technology, entry-level segment). Each 3D printed model was scanned and superimposed onto the reference master model and digital deviation analysis was performed to assess the trueness and precision calculated as root mean square (RMS). All data were statistically examined to obtain intra-group and inter-groups comparisons(*p* 0.05).

**Results:**

In both CM and DEM groups, SLA 3D printers* (*Vector 3SP and Form 3B) showed lower trueness error compared to DLP/LCD technologies (Asiga Pro 4K65, Anycubic Photon M3) (*p* < 0.001). In general, the entry-level printer (Anycubic Photon M3) showed the greatest trueness error (*p* < 0.001). Comparing CM and DEM models generated with the same 3D printer, statistically significant differences were found only for Asiga Pro 4k65 and Anycubic Photon M3 printers (*p* > 0.05). Concerning data of precision, the DLP technology (Asiga Pro 4k65) showed lower error compared to the other 3D printers tested. The trueness and precision errors were within the accepted clinical error for clear aligner manufacturing (< 0.25 mm), with the entry-level 3D printer nearly reaching this value.

**Conclusions:**

The accuracy of orthodontic models generated for clear aligners can be affected by different 3D printer technologies and anatomical characteristics of dental arches.

## Introduction

Clear aligners are becoming a prevalent aesthetic opportunity for orthodontic treatment [1]. The digital workflow for producing clear aligners is based on the orthodontic setup followed by 3D printing sequential digital models, each representing a single step of the programmed orthodontic movements. Afterward, clear aligners are produced by thermoforming a biocompatible transparent thermoplastic sheet using a vacuum thermoformer [2].

There are two principal clinical and managerial scenarios for dentists and orthodontists in the decision-making process for using clear aligners. The first is to refer to third-party companies with an available digital platform for the orthodontic setup and industrial equipment for clear aligners production, and the second is to establish in-office CAD-CAM workflow with dedicated equipment. Especially in the latter scenario, clinicians should be aware of the characteristics of different 3D printing systems and their capability and printing accuracy of anatomical models for clear aligners production, also considering that the market offers a wide range of 3D printers from entry-level to dental application systems.

Among many material processing processes, the Vat curing system is the most popular 3D printing technique for dental and orthodontic applications [3]. According to how the light source is used, 3D printers are categorized as stereolithography (SLA), digital light processing (DLP), and liquid crystal display (LCD), depending on how the liquid resin is cured in the Vat curing system [4]. Previous studies suggest that most of the available technologies could be appropriate for producing clear aligners [5–7]. Other well documented factors potentially affecting the accuracy of printed objects are model ortientation [8], layer thickness [9] and post-curing methods [10].

Even the anatomical characteristics of the printed objects could introduce some bias in the prototyping process. For example, a recent study would suggest some differences in the accuracy of prototyping models with aligned or crowded dentition and criticized that studies on this topic are generally performed on aligned dentition, which does not reflect the clinical pre-treatment scenario of orthodontic patients [7]. Surprisingly, no studies in the literature have comparatively investigated the accuracy of prototyped models, including diastema and edentulous areas. In this regard, it is crucial to validate various 3D printing methods and models for the orthodontic correction of diastemas or the management of prosthetic space.

The aim of the present study was to evaluate and compare the accuracy of orthodontic models featuring different clinical characteristics (crowded dentition, diastemas, and edentulous areas) prototyped with 3D printers featuring different technologies. Accuracy was defined according to the ISO standard 5725–1:1994, i.e., analyzing both trueness and precision of prototyped orthodontic models. The null hypotheses were the absence of significant differences in the values of trueness and precision among the models prototyped with different 3D printers and featuring different anatomical configurations.

## Materials and method

This study was carried out in accordance with the Helsinki Declaration on medical protocols and ethics, and was approved by the Institutional Ethical Committee of the University of Catania (protocol n. 153/2022/PO – A.M.D.A.).

### Digital reference models

Two mandibular dental scans (CS 3700, Carestream, Rochester, NY, USA; accuracy = 30 μm) were obtained respectively from 1) 20-year-old woman featuring anterior crowding, 2) 40-year-old woman featuring anterior diastema and edentulous spaces in the posterior region. Both patients had excellent oral hygiene with no caries, dental restorations, gingivitis, or periodontal disease. The two mandibular digital models, i.e. the model with crowded dentition (CM model) and the model with diastemas and edentulous spaces (DEM model) were used as digital reference models of the present investigation [7,14-17]. Signed informed consent was obtained from the two subjects recruited for intra-oral scan registrations.

**Fig. 1:**
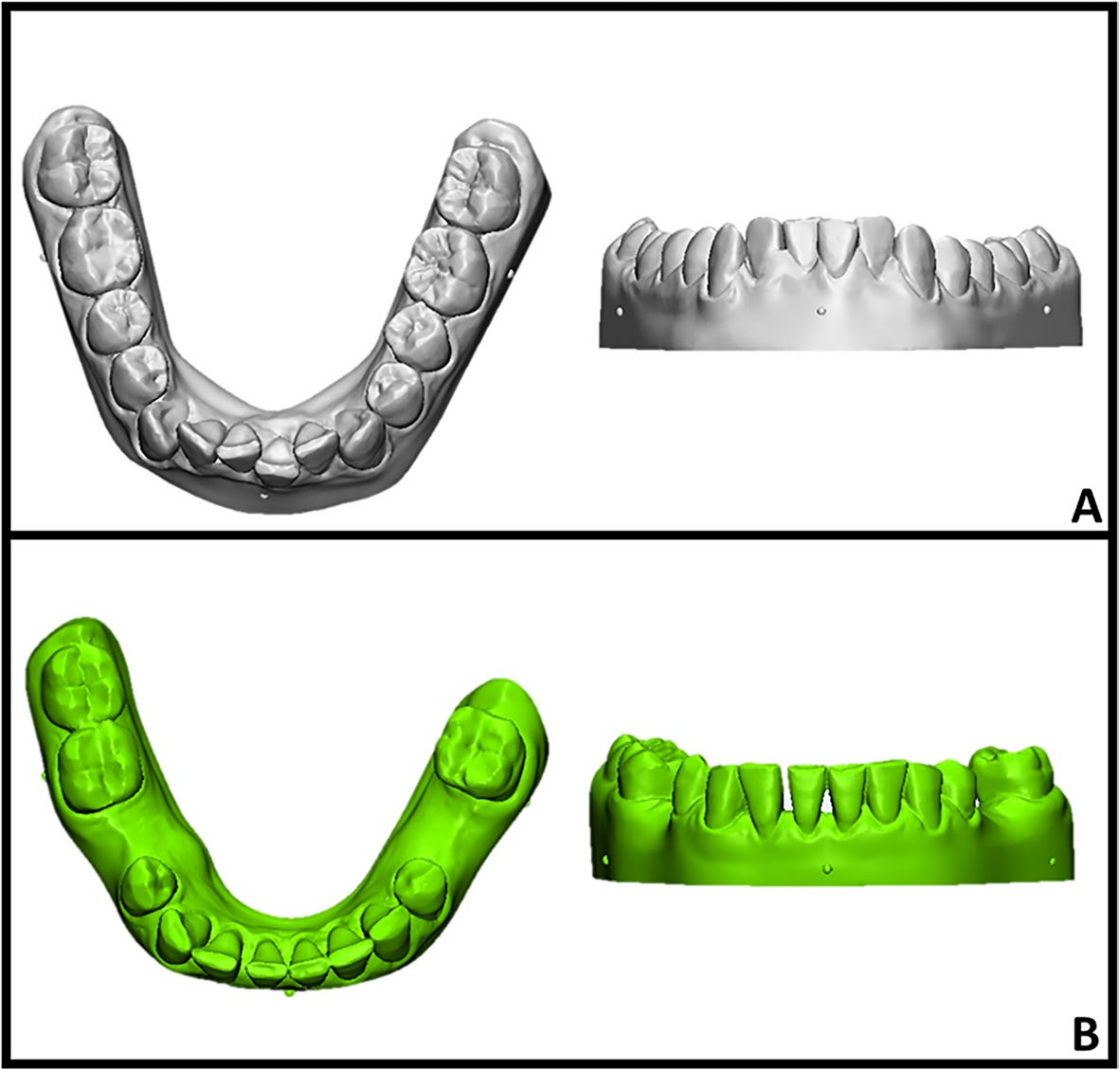
Master stereolithographic models (occlusal and frontal view): **A** crowded model (grey color); **B** model with diastemas and edentulous areas (green color)

The master models were imported into open-source 3D modeling software (Meshmixer 3.1.373; Autodesk, San Rafael, California) and optimized for 3D printing and subsequent digital overlay procedures. The model was designed using a basic horseshoe-shaped configuration. Three half-sphere markers were created along the vertical axis of the first molars (3 mm below the gingival margin) and the midline of the central incisors (5 mm below the gingival papilla). These points served as reference points for the preliminary point-based superimposition of the models and to generate a plane cut to exclude the printed base of the model from the surface deviation analysis [7] (Fig. 1).

### Models prototyping

**Fig. 2:**
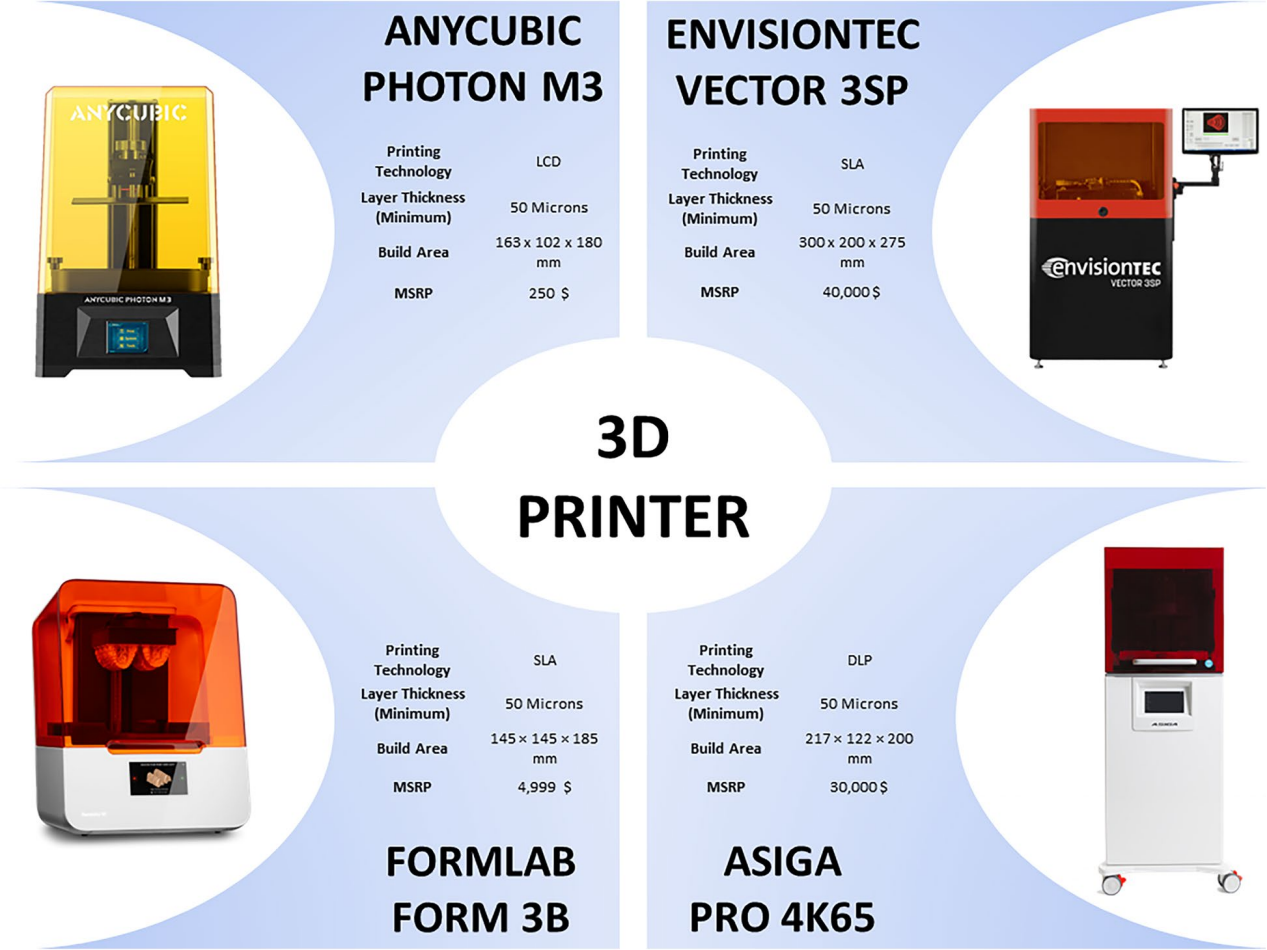
Characteristics of the 3D printers and layer thickness tested

The CM and DEM digital master models were printed according to four different printing protocols, using the resin, software and printing parameters recommended by the manufacturer [7]. Tested 3D Printers (Fig. 2): Anycubic Photon M3 (LCD Masked Technology, Entry-Level Segment; *Anycubic Technology Co*., Shenzhen, China), Form 3B (SLA-Laser Technology, Mid-Professional Segment; *Formlab*, Somerville, MA, USA), Asiga Pro 4K65 (SLA-DLP technology, highly professional segment; *Asiga*, Alexandria, Australia), Vector 3SP (SLA-Laser technology, industrial segment; *EnvisionTEC*, Dearborn, Mich). Slicing software: Photon Workshop software (*Anycubic Technology Co*., Shenzhen, China) for Anycubic Photon M3, PreForm software (*Formlab*, Somerville, MA, USA) for Form 3B, Asiga Composer software (*Asiga*, Alexandria, Australia) for Asiga Pro 4K65 and 3DPrinterOS (*EnvisionTEC*, Dearborn, MI, USA) for “Vector SP”. Slicing was done evenly, meaning each slice was separated at equal intervals. Photosensitive resins: Monocure 3D rapid model (Monocure PTY LTD, Berala, Australia), Formlab Model Resin (Formlab, Somerville, MA, USA), Asiga DentaFORM (Asiga, Alexandria, Australia), and E-Model 3D (EnvisionTEC, Dearborn, MI, USA) used for the Vector 3SP printer. All models were printed with the occlusal plane parallel to the build platform without using supports, and each print job produced a single model. Layer thickness was set at 50 μm since this value was considered the most balanced layer thickness to prototype orthodontic models for clear aligners [5, 7]. Ten models were generated for each 3D printer from CM and DEM digital models; therefore, 80 models were prototyped for the present investigation, 40 models for the CM group and 40 models for the DEM group.

The base of each model was marked to identify the print source and print job. The post-printing procedures were performed as follows: 2 separate immersion baths of 97% isopropyl alcohol (Faichim, San Giovanni Lupatoto—VR, Italy) using the Eurosonic®3D ultrasonic cleaner (Euronda Spa, Sandrigo – VI, Italy), air drying at room temperature for 30 s, and a cure cycle of 15 min for Monocure 3D rapid model, Formlab Model Resin, Asiga DentaFORM and 10 min for E-Model 3D Resin.

The curing process was carried out using curing machines supplied by the companies themselves: Anycubic Wash & Cure Machine (wavelength 385–405 nm; Anycubic Technology Co., Shenzhen, China), Form Cure machine (wavelength 385–405 nm; Formlab, Somerville, MA, USA), Asiga Flash (wavelength 385–405 nm; Asiga, Alexandria, Australia), and the Ultra/ Xtreme UV Light Curing Apparatus (wavelength wavelength 390—420 nm; EnvisionTEC, Dearborn, MI, USA).

### Registration of prototyped scanned models and digital models

**Fig. 3:**
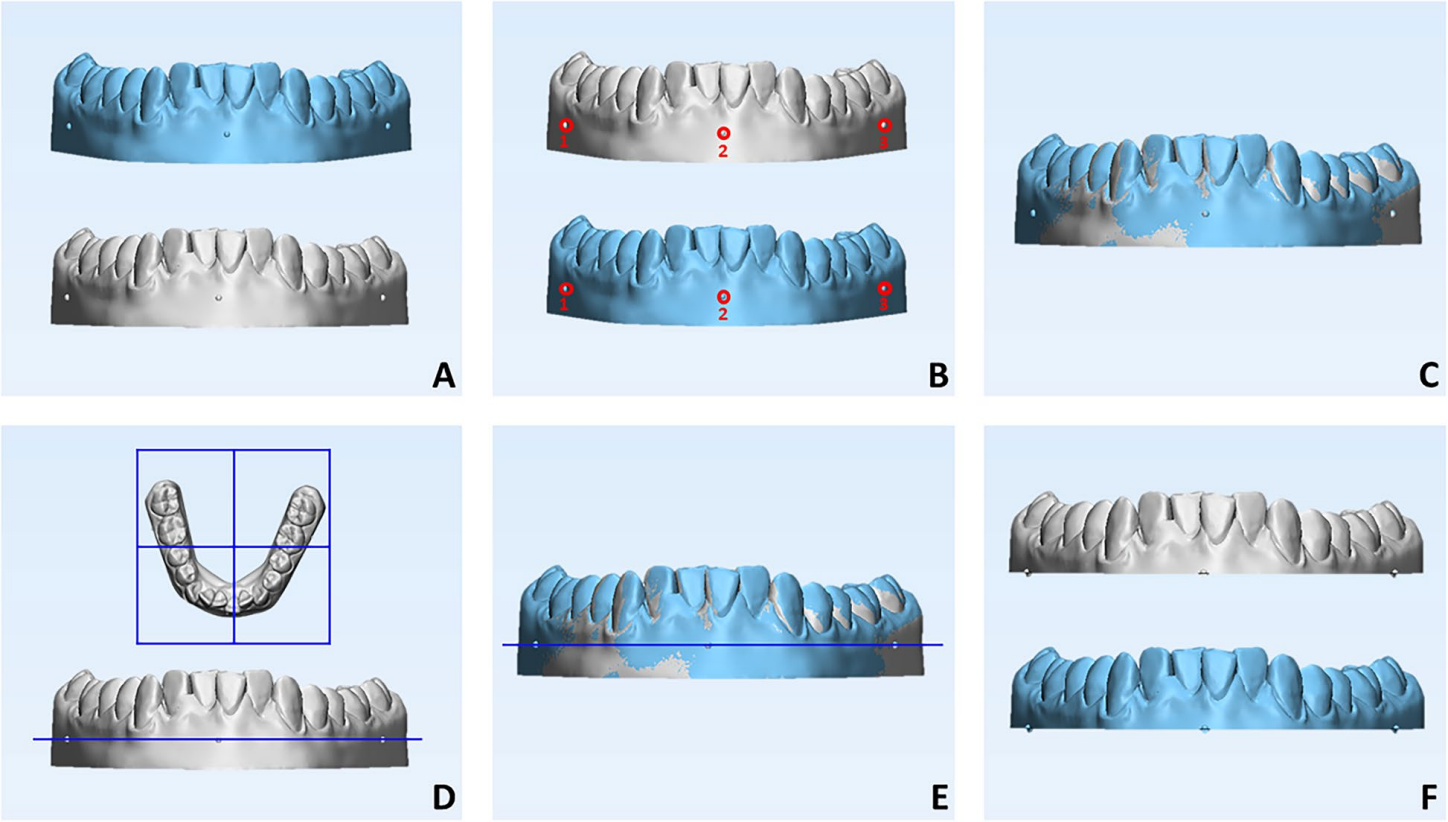
3D imaging technology involved for the superimposition between digital master model (light blue color) and scanned model (grey color). **A**-**B** point-based registration between the digital master model and the scanned prototyped model; **C** best-fit registration between the same models; **D**-**F** generation of the plane cut passing through the three reference points and used to remove the base of the initial master and scanned models

Each model printed in 3D was scanned using D2000 desktop scanner (3Shape, Copenhagen, Denmark; trueness = 5 μm) and the obtained STL files were exported to 3-Matic research software (vr. 13.0.0.188, Materialize, Leuven, Belgium) to perform the superimposition between each scanned model and the digital master model for the subsequent analysis of trueness. Firstly, a point-based registration was performed using the three half-sphere markers as a reference, followed by the final registration of the two models based on the automated best-fit algorithm (Fig. 3a-c). Then, a cutting plane was created that passes through the same reference points to eliminate the model base and, consequently, the distortion associated with removing the model from the print plane (Fig. 3d-f). The same procedure was used to superimpose the scanned prototyped models from the same group generated with the same 3D printer to analyze precision. For this purpose, five STL models obtained from the four 3D printers were randomly selected to perform ten intra-groups combinations for precision analysis.

### Deviation analysis

The STL files of the superimposed models were imported into Geomagic Control X software (3D Systems, version 2018.1.1, 3D Systems, USA) to calculate the distances between homologous surface points after the superimposition. The distances between the surface points of the two digital models were converted into root mean square (RMS), i.e., the mean value of the errors in comparing two datasets with the same coordinate system. RMS values represented trueness or precision data depending on the model used as a reference for the deviation analysis (original master model = trueness rating; 3D printed models generated with the same printer = precision rating).

**Fig. 4:**
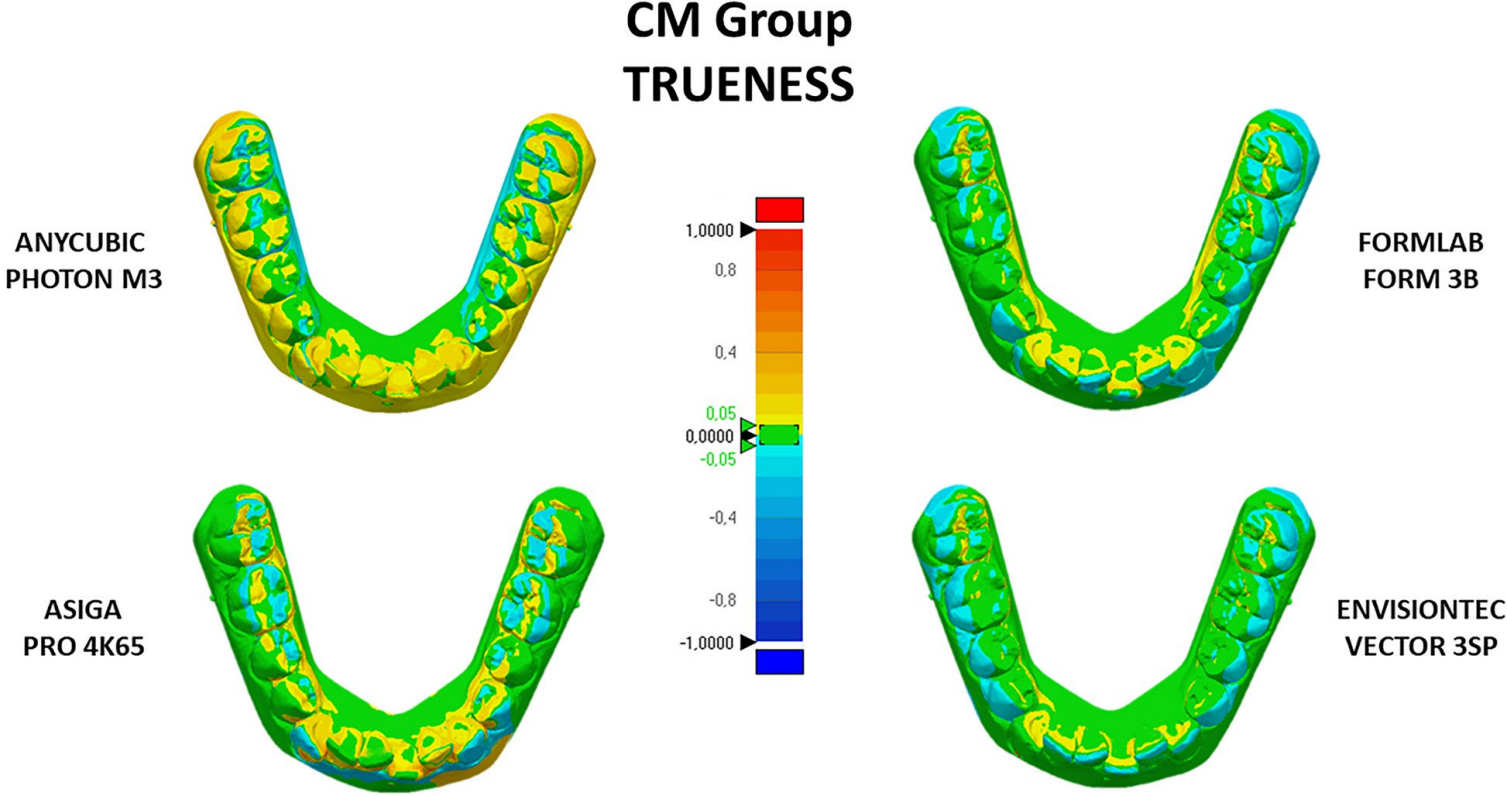
Color-coded maps obtained from the registration of the scanned printed crowded models (CM group) with the digital master model (analysis of trueness). Yellow-to-red fields = positive, turquoise-to-dark blue = negative, green = range of tolerance (± 0.05 mm)

**Fig. 5:**
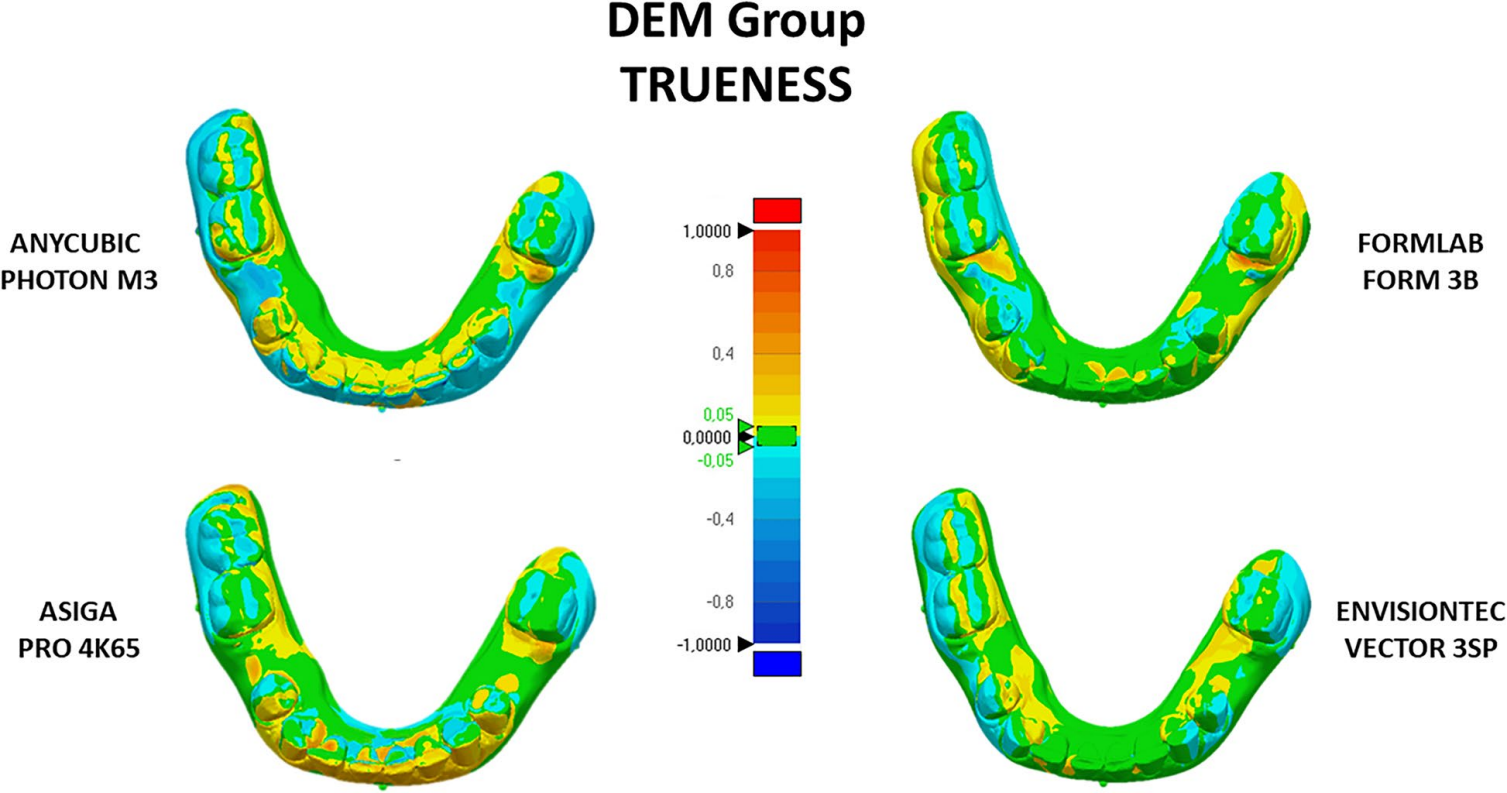
Color-coded maps obtained from the registration of the scanned printed models with edentulous areas and diastemas (DEM group) with the digital master model (analysis of trueness). Yellow-to-red fields = positive, turquoise-to-dark blue = negative, green = range of tolerance (± 0.05 mm)

The color-coded 3D map indicated when distance values were greater than the positive limits (yellow to red fields) or when the distance values were less than the negative limits (turquoise to dark blue). The tolerance range (green color) was set at ± 0.05 mm (Figs. 4 and 5). For clinical interpretation of the present data findings, the cut-off RMS value for the analysis of accuracy (trueness and precision) was set at 0.25 mm.

The entire workflow for digital analysis was computed by a single operator (V.R.) and the procedure (excluding the prototyping process) was repeated two weeks later to analyze intra-observer variability and method error. A second operator (A.L.G.) also performed the procedure to assess reliability among observers.

### Statistical analysis

Sample size calculation was assessed using preliminary unpublished data. In this regard, the RMS values obtained from the CM and DEM groups were compared using independent sample t-test, with an alpha error set to 0.05. The analysis suggested that five models in each group were required to reach 95% power to detect an average RMS difference of 0.015 mm between the two groups. However, we decided to tested 10 models for each group, increasing the power of the available data.

Shapiro–Wilk test and the Levene test were used to calculate normal distribution and equality of data variance. One-way analysis of variance (ANOVA) was used to compare the trueness and precision of printed models; the Scheffè's method was used to perform Post-hoc multiple comparison [16]. Independent Student's t-test was used for comparatively analysis of accuracy and precision between CM and DEM groups. The intraclass correlation coefficient (ICC; model = 2-way mixed effects, type = single measure, definition = absolute agreement) was performed to calculate intra-examiner and inter-examiner reliability, while method error was calculated using the Dahlberg formula. Data were analyzed using SPSS® version 24 Statistics software (IBM Corporation, 1 New Orchard Road, Armonk, New York, USA).

## Results

Statistically significant differences were found in the RMS values of trueness for dental models printed with the tested 3D printers (*p* < 0.001) (Table 1). The RMS values recorded were significantly lower for SLA 3D printers (CM group: Form 3B, 0.082 ± 0.009 mm; Vector 3SP, 0.079 ± 0.001 mm; DEM group: Form 3B, 0.083 ± 0.008 mm; Vector 3SP, 0.080 ± 0.008 mm) compared to DLP/LCD 3D printers (CM group: Asiga Pro 4k65, 0.155 ± 0.012 mm; Anycubic Photon M3, 0.214 ± 0.015 mm; DEM group: Asiga Pro 4k65, 0.154 ± 0.019 mm; Anycubic Photon M3, 0.208 ± 0.017 mm) in both CM and DEM groups (Table 1). Concerning the type of models printed, no significant differences were found comparing the RMS values recorded between CM and DEM models, for each 3D printer tested (*p* > 0.05) (Table 2).

**Table 1:** Comparison of Root mean-squared (RMS) values of trueness of models with diastema and edentulous spaces (DEM) and models with crowded dentition(CM) prototyped with different 3D printers

3D Printers	N	Groups	RMS (mm)	SD	95% C.I		Significance
					**Lower limit**	**Upper limit**	
Asiga Pro 4k65 (a)	10	DEM	0,154 (b,c,d)	0,019	0,140	0,168	*p* < 0.001
Photon M3 (b)	10	DEM	0,208 (a,c,d)	0,017	0,196	0,221	
Form 3B (c)	10	DEM	0,083 (a,b)	0,008	0,077	0,090	
Vector 3SP (d)	10	DEM	0,080 (a,b)	0,006	0,075	0,086	
Asiga Pro 4k65 (a)	10	CM	0,155 (b,c,d)	0,012	0,145	0,164	*p* < 0.001
Photon M3 (b)	10	CM	0,214 (a,c,d)	0,015	0,203	0,225	
Form 3B (c)	10	CM	0,082 (a,b)	0,009	0,075	0,088	
Vector 3SP (d)	10	CM	0,078 (a,b)	0,010	0,070	0,086	

Significance set at *p* < 0.05 and based on one-way analysis of variance (ANOVA) and Tukey’s post-hoc comparisons tests a, b, c, d = identifiers for post-hoc comparisons tests

*N* Sample number, *SD* Standard deviation, *C.I* coefficient interval

**Table 2:** Comparison of Root mean-squared (RMS) values of trueness between models with diastema and edentulous spaces (DEM) and models with crowding (CM) for each 3D printer tested

3D Printers	Groups	N	RMS (mm)	SD	RMS Difference (mm)	SE Difference	Significance
Asiga Pro 4k65	DEM	10	0,154	0,019	0,001	0,007	NS
	CM	10	0,155	0,012			
Photon M3	DEM	10	0,208	0,017	0,005	0,007	NS
	CM	10	0,214	0,015			
Form 3B	DEM	10	0,083	0,008	0,001	0,004	NS
	CM	10	0,082	0,009			
Vector 3SP	DEM	10	0,080	0,006	0,002	0,004	NS
	CM	10	0,078	0,010			

*N* Sample number, *SD* Standard deviation, *Diff* Difference, *NS* Not significant. Significance set at *p* < 0.05 and based on unpaired Student's t test

Statistically significant differences were found in the RMS values of trueness for dental models printed with the tested 3D printers (*p* < 0.001) (Table 3). The RMS values were significantly lower for the Asiga Pro 4k65 (CM group: 0.039 ± 0.012 mm; DEM group: 0.040 ± 0.013 mm) compared to both SLA 3D printers (CM group: Form 3B, 0.049 ± 0.014 mm; Vector 3SP, 0.050 ± 0.014 mm; DEM group: Form 3B, 0.051 ± 0.016 mm; Vector 3SP, 0.048 ± 0.018 mm;) and entry-level 3D printer (CM: Anycubic Photon M3, 0,039 ± 0.012 mm; CM group: Anycubic Photon M3, 0,040 ± 0.013 mm) (*p* > 0.05) (Table 3). No significant differences were found comparing the RMS values recorded between CM and DEM models, for each 3D printer tested (*p* > 0.05) (Table 4).

**Table 3:** Comparison of Root mean-squared (RMS) values of precision of models with diastema and edentulous space (DEM) and models with crowding (CM) prototyped with different 3D printers

3D Printers	N	Groups	RMS (mm)	SD	95% C.I		Significance
					**Lower limit**	**Upper limit**	
Asiga Pro 4k65 (a)	10	DEM	0,040 (b)	0,013	0,031	0,050	*p* < 0.001
Photon M3 (b)	10	DEM	0,095 (a,c,d)	0,019	0,081	0,108	
Form 3B (c)	10	DEM	0,051 (b)	0,016	0,039	0,063	
Vector 3SP (d)	10	DEM	0,048 (b)	0,014	0,036	0,060	
Asiga Pro 4k65 (a)	10	CM	0,039 (b)	0,012	0,030	0,048	*p* < 0.001
Photon M3 (b)	10	CM	0,100 (a,c,d)	0,025	0,082	0,118	
Form 3B (c)	10	CM	0,049 (b)	0,014	0,038	0,059	
Vector 3SP (d)	10	CM	0,050 (b)	0,014	0,040	0,060	

Significance set at *p* < 0.05 and based on one-way analysis of variance (ANOVA) and Tukey's post-hoc comparisons tests; a, b, c, d = identifiers for post-hoc comparisons tests

*N* Sample number, *SD* Standard deviation, *C.I* Coefficient interval

**Table 4:** Comparison of Root mean-squared (RMS) values of precision between models with diastema and edentulous spaces (DEM) and models with crowding (CM) for each 3D printer tested

**3D Printers**	**Groups**	**N**	**RMS**	**SD**	**RMS Difference (mm)**	**SE Difference**	**Significance**
			**(mm)**				
Asiga Pro 4k65	DEM	10	0,040	0,013	0,001	0,005	NS
	CM	10	0,039	0,012			
Photon M3	DEM	10	0,095	0,019	0,005	0,01	NS
	CM	10	0,100	0,025			
Form 3B	DEM	10	0,051	0,016	0,002	0,007	NS
	CM	10	0,049	0,014			
Vector 3SP	DEM	10	0,048	0,014	0,002	0,006	NS
	CM	10	0,050	0,014			

*N* Sample number, *SD* Standard deviation, *NS* Not significant. Significance set at *p* < 0.05 and based on unpaired Student's t test

Concerning the reliability of the digital procedure, ICC tests showed no difference between the two readings with an excellent correlation ranging from 0.939 to 0.951 for intra-observer reliability (and 0.905 to 0.928 for inter-observer reliability. According to the Dahlberg formula, the reported method error was identified by the fifth decimal place of the RMS values for accuracy and precision.

## Discussion

To the best of our knowledge, this is the first study in the literature that evaluates the accuracy of different 3D printing technologies by testing orthodontic models with opposite clinical conditions, i.e. misaligned models and models with diastemas and edentulous spaces. In this regard, there is an urgent need to analyze all the potential sources of error in the prototyping process of orthodontic models, especially if we consider the importance of aligners fitting in real dentition for the expression of the programmed forces. The authors maintain that the data reported in the present study will provide useful information for those clinicians who have decided to approach the in-office production of clear aligners. Also, the authors' concerns regarding clear aligner therapy are limited to the accuracy values of the 3D printed models, which represent the first step of clear aligner manufacturing. The physico-chemical and biomechanical characteristics of the clear aligners [11] were all beyond the scope of the present investigation.

Concerning the methodology applied for the deviation analysis of the models, the present study has two main strengths: first, we excluded from the analysis most of the gingival surface of the original typodont which represents a non-target area in orthodontic models finalized for clear aligners production [12]; second, we used specific reference points outside the dental region to perform the first point-based registration and to consistently reproduce the horizontal plane cut [13]. Finally, the cut-off RMS value for the analysis of accuracy was set at 0.25 mm since this value is in the range of maximum tooth movement planned per each aligner [9]; thus, prototyped dental models must report an accuracy below this range for clinical application of clear aligners.

In this regard, all models generated with the 3D printers tested in this study would be adequate to produce clear aligners as the recorded trueness and precision errors (RMS value) was within the clinically accepted error of 0.25 mm in both the CM and in the DEM groups. However, some differences were found between 3D printing technologies and study groups and should be considered for in-office application of model production for clear aligners.

In both groups, SLA 3D printers (Form 3B and Vector 3SP) showed lower trueness error compared to DLP or LCD 3D printers (Anycubic Photon M3 and Asiga Pro 4k65); even if we limit the comparison between the two lab-professional 3D printers, SLA technology (Form 3B) performed better than DLP technology (Asiga Pro 4k65). Comparing data of trueness error between CM and DEM groups, the models generated with Anycubic Photon M3 and Asiga Pro 4k65 showed similar values of RMS in presence of crowded dentition and spaces; similarly, no significant differences were found between CM and DEM for Form 3B and Vector 3SP printers. Both findings obtained from 3D printers comparisons and models comparisons would suggest that laser-based curing technology could be more accurate than DLP-based technology [14].

The color-coded maps clearly showed the different behaviors of SLA and DLP/LCD 3D printers in generating models with crowding (Fig. 4) and models with interdental spaces (Fig. 5). In both cases, there is greater mismatching between the digital model and the prototyped model with Anycubic Photon M3 and Asiga Pro 4k65. Such mismatch is mostly visible in the occlusal surfaces and in the inter-proximal surfaces of the teeth involved in the crowded areas and in those contiguous to the spaces. This aspect would confirm that SLA printing technology could be the best choice when the aim is to obtain better definition of printed objects, in particular in those areas characterized by undercuts, complex morphologies (for example, occlusal surfaces) [15], but also thin surfaces such as the mesial and distal surface of teeth with diastema or spaces, compared to DLP technology. The explanation may be that SLA 3D printers use a laser-beam to cure the resin and it is not dependent by x–y resolution; instead, DLP printers build the object by curing the resin layer by layer and could generate subtle artifacts at layer edges that look like “staircase steps” due to the pixelated illumination generated from the light source (projector or LCD screen for LCD 3D printers).

From the clinical perspective, since each aligner is shaped on dental surfaces, models prototyped with SLA 3D printers can produce aligners with greater fitting in the inter-proximal surfaces, increasing the effectiveness of the programmed movement for alignment (CM models) and for space closure or anchorage management (DEM models). However, this assumption must be confirmed by clinical studies comparing the effectiveness of in-office clear aligners produced from different printing systems. Also, future studies assessing extreme anatomical conditions such as serious rotated teeth or infra-occlusion are encouraged.

Concerning precision variable, the tested 3D printers produced a repeated error below the clinical cutoff value of 0.25 mm. The Asiga 4k65 showed slightly lower RMS values compared to the SLA 3D printers tested (Form 3B and Vector 3SP). In this regard, the assumption supporting this finding is that DLP devices feature a less complex pattern of printing process (layer by layer light-source curing process) compared to the SLA technology (simultaneous interaction between laser beam and guide mirrors) [13]. However, the differences found in this study between DLP and SLA printers are far from being considered clinically relevant.

The Anycubic Photon M3 showed lower accuracy compared with other 3D printers. This findings would confirm the assumption that since entry-level printers are assembled with low-budget hardware components and modules they can be exposed to significant error in repeated processes [7]. In particular, models printed with Anycubic Photon M3 showed a trueness error that was close to the threshold value for producing clear aligners (0.25 mm). If we consider that the reliability of the planned tooth movement with clear aligners is influenced by the cumulative error generated in each prototyped model [7, 11, 16], the present findings suggest that clinicians should be careful when using entry-level 3D printers and should limit their applications to minor orthodontic movements (for example, orthodontic refinement).

In this study, all orthodontic models were prototyped using the resin, settings and post-curing devices dedicated or recommended for each 3D printer. This choice was based on the necessity to test the printing devices at the best of their claimed performance. Future studies could address the evaluation of different printing and post-curing settings using one dental resin material, to provide new evidences on the printing accuracy of different devices [4, 17].

## Conclusion

- SLA technology would produce orthodontic models with greater accuracy compared to DLP/LCD technologies. According to the color-coded map, the areas mostly affected by trueness error with DLP/LCD 3D printers were the occlusal surfaces and the interpromixal surfaces of crowded teeth or spaced teeth.
- All models generated with the 3D printers tested would be adequate to produce clear aligners as the recorded trueness error (RMS value) was within the clinically accepted error of 0.25 mm.
- Caution should be taken with the usage of entry-level 3D printers since the RMS values of trueness and precision error was close to the clinical threshold.

## Data Availability

The datasets used and analysed during the current study are available from the corresponding author on reasonable request.

## Abbreviations

CAD-CAM: Computer aided design – Computer aided manufacturing
SLA: Stereolithography
DLP: Digital light processing
LCD: Liquid crystal display
CM model: Model with crowded dentition
DEM model: Model with diastemas and edentulous spaces
RMS: Root Mean Squared
